# Competitive Promoter-Associated Matrix Attachment Region Binding of the Arid3a and Cux1 Transcription Factors

**DOI:** 10.3390/diseases5040034

**Published:** 2017-12-10

**Authors:** Dongkyoon Kim, Christian Schmidt, Mark A. Brown, Haley Tucker

**Affiliations:** 1 Molecular Biosciences, Institute for Cellular and Molecular Biology, University of Texas at Austin, Austin, TX 78715, USA; kimdk1@atreca.com (D.K.); Christian.schmidt@iap.fraunhofer.de (C.S.); 2 Atreca, Inc., Redwood City, CA 94063, USA; 3 Department of Biomaterials and Healthcare, Division of Life Science and Bioprocesses, Fraunhofer-Institute for Applied Polymer Research (IAP), 14476 Potsdam-Golm, Germany; 4 Department of Clinical Sciences, Colorado State University, Fort Collins, CO 80523, USA; Mark.Brown@colostate.edu

**Keywords:** immunoglobulin heavy chain, Arid3a, NF-µNR, matrix-attachment region (MAR), transactivation

## Abstract

Arid3a/Bright/Dril1 is a B cell-specific transactivator that regulates immunoglobulin heavy chain (IgH) gene transcription by binding promoter and enhancer-associated matrix attachment regions (MARs) within the IgH gene locus. Promoter MAR-mediated Arid3a transactivation is antagonized by direct competition of MAR binding by Cux1/CDP—a ubiquitously expressed repressor originally termed NF-μNR. We report that the NF-μNR complex includes Arid3a in B cells but not in non-B cells through mobility shift assays. The binding activity of NF-μNR and Arid3a in B cells is reciprocally altered during the cell division cycle and by the B cell mitogen lipopolysaccharide LPS. LPS treatment had no effect on Arid3a localization but increased its total abundance within the nucleus and cytoplasm. We show that this increased level of Arid3a is capable of displacing Cux from the MARs to facilitate IgH gene transcription. Finally, we showed that the MARs (termed Bf150 and Tx125) associated with the V_H_1 rearranged variable region expressed in the S107 murine plasmacytoma, can repress reporter gene transcription in non-B cells and that they can relieve the repression mediated by Eμ enhancer in B cells. These results have significant implications for early human development and demonstrate that MARs in IgH locus, NF-µNR and Arid3a regulate IgH gene expression in a concerted fashion. This paves the way for future studies examining the misregulation of this pathway in pediatric disease.

## 1. Introduction

**Figure 1:**
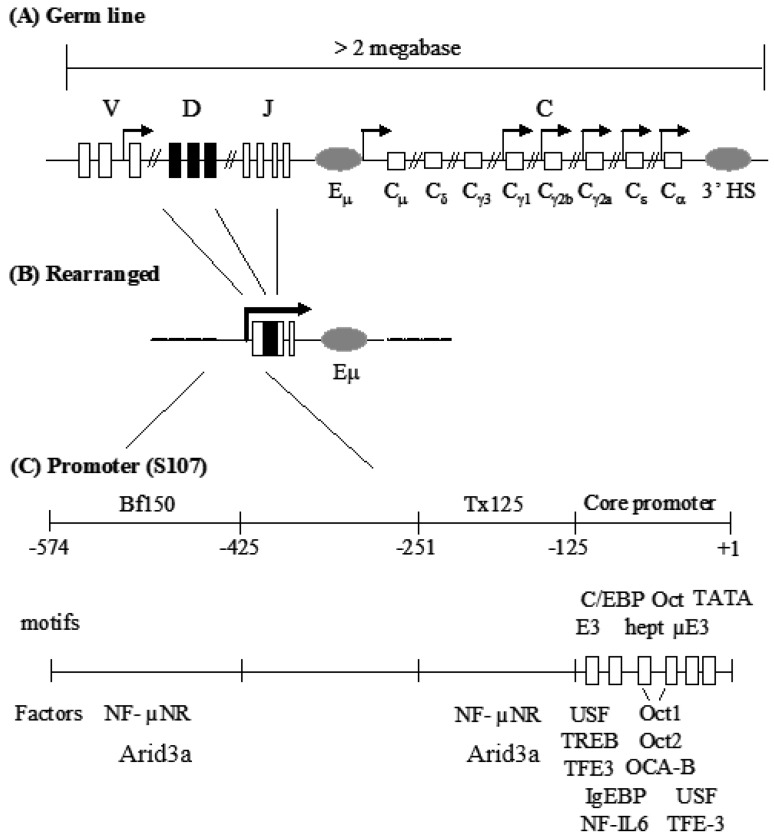
Mouse IgH locus and the S107 V_H_1 promoter region. (**A**) IgH gene locus consists of gene segment clusters in the germ line. Boxes indicate exons and gray ovals are enhancers. Arrows indicate germ-line transcription; (**B**) Gene rearrangement assembles a functional VDJ exon and juxtaposes the V_H_-associated promoter with the intronic enhancer, Eµ; (**C**) The promoter of the V_H_1 variable gene of the S107 family is composed of a core promoter and two MARs. Closed triangles and open boxes represent cis protein binding sites within MARs or core promoter, respectively. Transcription factors are shown under the motifs to which they bind.

Immunoglobulin heavy chain (IgH) gene transcription has served as a model system to study tissue-specific and developmental stage-specific gene regulation. One of the prominent features of IgH gene regulation is that numerous regulatory elements are scattered across the expansive *IgH* locus (Figure 1; Figure S1). These include the V_H_ promoter, the intronic enhancer (Eμ) and the 3’ enhancers [1,2,3,4]. These elements are composed of various transcription factor binding motifs which, in some cases, are flanked or are proximal to matrix attachment regions (MARs) [5,6]. Considerable effort has focused on elucidating the functions of each of these elements. For example, in some contexts, either the promoter or Eμ, alone, is sufficient for tissue-specific expression of *IgH*. Yet the combination of the two elements achieved by somatic VDJ recombination leads to the high levels of transcription required to achieve antibody levels necessary for defense [6,7]. In addition, the contribution of critical motifs within each of these elements, such as the octamer within the promoter and Eμ, has been studied extensively [8].

In spite of intense interest in the V_H_ core promoter, far less is known about the promoter-associated MARs. The MARs (operationally termed Bf150 and Tx125 and residing ~150 and ~125 bp upstream of the V_H_1 promoter) were reported to be required for maximal transcriptional induction of the murine S107 V_H_ locus by mitogens and certain cytokines [9]. These MARs contain specific binding sites for Arid3a and its antagonist, CDP/Cux [10,11]. Since Arid3a is expressed primarily in B cells, these data raised the possibility that Bf150 and Tx125 might function in a lineage-specific manner. Stable transfection of a reporter containing the V_H_1 core along with Bf150 and Tx125 into a plasma B cell line (J558) achieved significant levels of transcription that could be further stimulated by Arid3a [10]. However, Arid3a could not transactivate the reporter through a concatamer of Bf150. These and related experiments led to the speculation that the appropriate arrangement or “germline” context of these elements is required for Arid3a to properly engage and activate them. However, a transgenic study of S107 transcription argued that Bf150 and/or Tx125 were dispensable for V_H_1 activation and that the core promoter region was sufficient to confer lymphocyte-specific expression [12]. Therefore, the roles of the Bf150 and Tx125 MARs in V_H_1-mediated transcription remained unclear.

Eµ, residing in the intron between VDJ and Cµ, is composed of core enhancer and the flanking MARs [6]. The Eµ is known to be sufficient for lymphoid-specific expression of IgH gene [13]. However, Aguilera et al. showed that some B cell lines maintain high expression of endogenous IgH locus with Eµ deletion, implying the existence of other functionally redundant activating elements in the IgH locus [14]. Meanwhile, Kaplan et al. demonstrated that Arid3a interacts Eµ-flanking MARs and transactivates flanking genes [10].

EMSA experiments demonstrated that Bf150 and Tx125 produce two major DNA-protein complexes in B cells: An Arid3a-containing complex and a NF-μNR-containing complex. It was proposed [9,15] that Arid3a and NF-μNR compete for binding to common “P sites” within each of these MARs, with the balance leading to activation or repression, respectively. Consistent with this model, CDP/Cux was identified as the functional component of NF-μNR, and competition with CDP/Cux abrogated Arid3a-DNA binding [15]. In several cases, CDP/Cux was documented to function by competing with activators for DNA binding sites [9,16]. In other cases, CDP/Cux was implicated to actively repress transcription by recruiting HDAC1 [17]. The activity of CDP/Cux is regulated during the cell cycle, as specific DNA binding was restricted to the G_1_/S transition and S phase [18]. However, in several cases, CDP/Cux DNA binding activity was shown to disappear as cells become fully differentiated [19].

An added complexity that was not considered in previous studies is the contribution of the related ARID transcription factor, Arid3b/Bdp. Although poorly characterized, the single study published on this Arid3a paralog demonstrated that in vitro translated Arid3b could also bind to bf150 and Tx125 [20]. We showed previously that Arid3b interacts with Arid3a to modulate its localization [21,22]. Yet it remained unclear as to whether Arid3a-Arid3b heteromers bind to the promoter-associated MARs, or if their homomeric and Arid3a heteromeric complexes serve redundant or specific functions.

In this study, we have investigated features and components of the Arid3a and NF-μNR complexes through EMSA/super-shift assays in order to better understand the function of promoter-associated MARs in IgH transcription. We demonstrate that these complexes are sensitive to cell cycle progression and to mitogen stimulation. We also employed a non-B cell transcription assay to examine the effect of Arid3a on the promoter- and enhancer-associated MARs in the absence of other B cell specific regulators.

## 2. Materials and Methods

All experiments for this study were performed in triplicate.

### 2.1. Cell Culture

M12.4, a mouse lymphoma [23], and BCL_1_, mouse leukemia [24] cells were cultured in RPMI medium supplemented with 10% fetal calf serum (FCS). The adherent cell lines, Cos-7 and NIH 3T3, were grown in Dulbecco modified Eagle medium (DMEM) supplemented with 10% FCS under standard conditions.

To examine the starvation effect, BCL_1_ cells were grown in RPMI complete medium for 3 days with or without the daily supplement of fresh medium. To stimulate BCL_1_ cells with LPS, the cells were grown in RPMI complete medium supplemented with 10 µg/mL of LPS for 2–3 days.

### 2.2. Transfections and Retroviral Transduction

NIH3T3-Arid3a stable lines were established using the Phoenix retroviral system according to published protocols (https://web.stanford.edu/group/nolan/_OldWebsite/retroviral_systems/phx.html) as previously described [21].

Luciferase-expressing NIH 3T3 stable cell lines (NIH 3T3 5′ MAR-Luc and NIH 3T3 5′ MAR-Luc-Eµ) were established by co-transfecting NIH 3T3 cells with a neomycin-resistant gene-bearing mini vector and the pGL3-5′ MAR construct using FuGene6 transfection reagent (Roche Diagnostics). Neomycin-resistant luciferase-positive clones were selected with 600 μg/mL of G418 for 12 days. The 5′ MAR luciferase-expressing NIH 3T3 stable cell lines were then transiently transfected with Arid3a constructs using FuGene6 transfection reagent. Briefly, 1.5 × 10^5^ to 3 × 10^5^ cells were cultured overnight in six-well plates containing 2 mL of medium. The cells were transfected with 0.3–1 μg of plasmid DNA and cultured for 24–48 h.

### 2.3. Nuclear Protein Extraction

Cytoplasmic and nuclear extracts were prepared according to the method of Johnson et al. [25]. B cells (~1 × 10^7^) were collected, washed twice with PBS, and pellets were suspended in 100 μL of sucrose buffer I-A (100 mM Tris-Cl, pH 8.0; 0.32 M sucrose; 3 mM CaCl_2_; 2 mM Magnesium acetate; 0.1 mM EDTA; 1 mM DTT; 0.5 mM PMSF and 0.5% NP-40) supplemented with 0.1 μL of protease inhibitor cocktail (Sigma, St. Louis, MO, USA). Nuclei were separated from the soluble cytoplasmic fraction by centrifugation at 2000 rpm for 2 min (Fisher Scientific, Model 59-A, Hampton, NH, USA). Nuclei were suspended in 20 μL of low salt buffer (20 mM HEPES, pH 7.9; 25% glycerol; 1.5 mM MgCl_2_; 20 mM KCl; 0.2 mM EDTA; 0.5 mM DTT and 0.5 mM PMSF), and then 20 μL of high salt buffer (20 mM HEPES, pH 7.9; 25% glycerol; 1.5 mM MgCl_2_; 800 mM KCl; 0.2 mM EDTA; 1% NP-40; 0.5 mM DTT and 0.5 mM PMSF) were added dropwise. Then, the extract was diluted with 100 μL of diluent (25 mM HEPES, pH 7.6; 25% glycerol; 0.1 mM EDTA and 0.5 mM PMSF). The mixture was centrifuged at 12,000 rpm for 15 min at 4 °C (Eppendorf, 5417R Hamburg, Germany), and the supernatant was used for EMSA.

### 2.4. Electrophoretic Mobility Shift Assays (EMSA)

In vitro DNA binding reactions were conducted in a total volume of 25 μL of binding buffer (10 mM HEPES, pH7.9; 10% glycerol (*v*/*v*); 50 mM NaCl; 0.5 mM EDTA; 0.02% Tween-20; 80 ng/μL poly-(dI/dC) and 0.25 mM PMSF), containing 2 μg of nuclear extract and ~80,000 cpm of Arid3a-specific probe: 5’-end labeled, gel-purified fragments spanning either of the V_H_ S107 promoter-associated MARs (Bf150 or Tx125) as previously described [26]. Samples were incubated for 20 min at room temperature and then resolved on 4% polyacrylamide gels.

For the super-shift assays, indicated antibodies or antisera were added to the above binding reaction following probe-extract incubation. Samples were placed on ice for 30 min before loading onto gels.

### 2.5. Cell Cycle Synchronization and Cell Cycle Analysis

To synchronize the cell cycle at G_1_/S phase, BCL_1_ cells were grown in RPMI medium supplemented with 0.5% FCS for 18 h and then shifted to RPMI medium supplemented with 10% FCS and 1 mg/mL of aphidicolin (Sigma) for 16 h. The cells were washed with phosphate-buffered saline (PBS) to remove the cell cycle inhibitor and grown in RPMI medium supplemented with 10% FCS. B cells were harvested every 2 h, fixed in ethanol, washed with PBS, incubated for 30 min with 10 μL of 50 μg/mL propidium iodide (Sigma) and 10 μL of 10 mg/mL RNase A, and then analyzed by flow cytometry for DNA content.

### 2.6. In Vitro Transcription/Translation

Arid3a was expressed using the TNT Coupled Transcription/Translation Systems (Promega, Madison, WI, USA) following the manufacturer’s instructions. Protein integrity and level was assessed by anti-Arid3a Western blotting.

### 2.7. Luciferase Assays

NIH 3T3 5′ MAR-Luc cells were transfected with pBabe-Arid3a wild type. Twelve or 24 h later, the luciferase activity was measured within 10 μg of whole-cell lysate using the luciferase reporter assay system (Promega) according to the manufacturer’s instructions. To evaluate the effect of Arid3a on luciferase reporter gene activity, we calculated the relative luciferase activities of Arid3a-expressing cells compared with that of NIH 3T3 5′ MAR-Luc.

## 3. Results

### 3.1. Components of the Arid3a and NF-μNR Complexes in B Cells

In previous studies, two major DNA binding complexes in B cells have been detected on the two MARs, Tx125 and Bf150, upstream of the IgH promoter [9,27]. NF-μNR forms a slowly migrating complex, and its functional component, CDP/Cux, is associated with IgH repression [17]. Arid3a forms a more rapidly migrating complex which is associated with locus activation. None of the previous studies, however, have directly compared the relative binding of Tx125 and bf150, nor have they considered the potential contribution of Arid3b.

**Figure 2:**
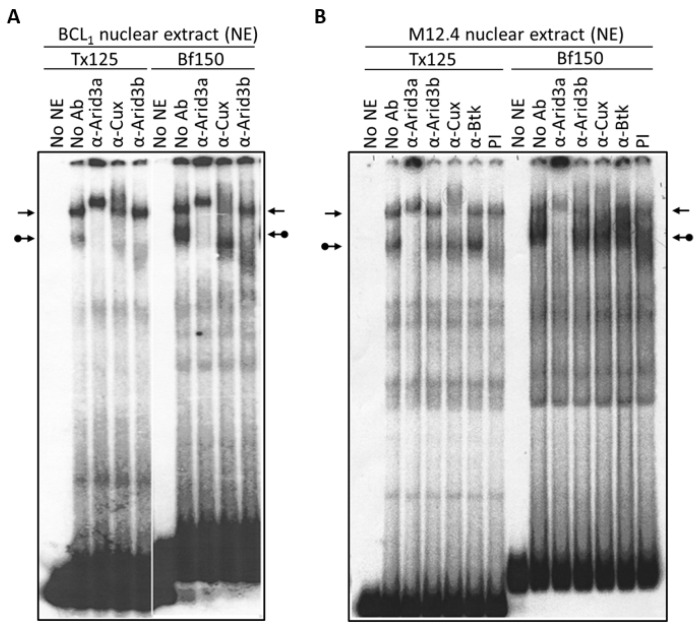
NF-μNR and Arid3a bind to V_H_1 promoter-associated MARs Tx125 and Bf150 with different affinities. BCL_1_ (**A**) and M12.4 (**B**) nuclear extracts were prepared and subjected to EMSA using ^32^P-labeled Bf150 or Tx125 fragments as probes. Super-shift assays were performed with antisera or mAB (α) listed above the corresponding lanes. Plane arrows indicate the mobility of the NF-μNR complex; arrows with balls, the Arid3a complex. No NE: free probe, PI: pre-immune serum.

**Figure 3:**
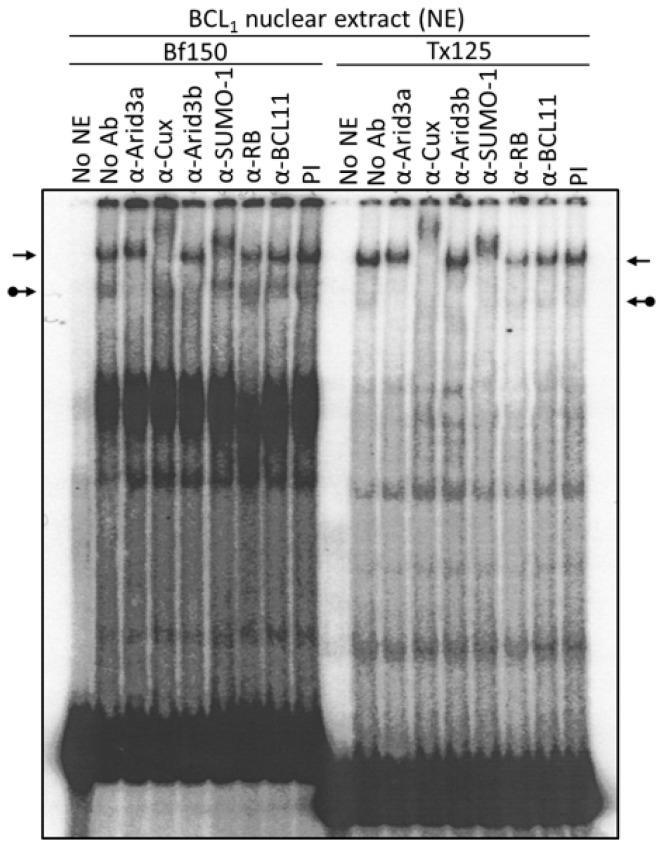
Arid3a and NF-μNR complexes with promoter-associated MARs are composed of heterogeneous proteins. BCL_1_ nuclear extract was prepared and subjected to EMSA using ^32^P-labeled Bf150 or Tx125 fragments as probes. Super-shift assays were performed with antisera or mAb (α) listed above the corresponding lanes. Plane arrows indicate the mobility of the NF-μNR complex; arrows with balls, the Arid3a complex. No NE: free probe, PI: pre-immune serum.

To re-examine these issues, we prepared nuclear extracts from the mature B cell lines, BCL_1_ and M12.4, and performed EMSA. As shown in Figure 2, the Arid3a-containing complex has a relatively higher affinity for Bf150 than for Tx125. Conversely, the NF-μNR complex exhibits higher affinity for Tx125 than for Bf150. The differential affinity of Arid3a and NF-μNR were observed in both M12.4 and BCL_1_ cells (Figure 2). In addition, we consistently observed that anti-Cux antiserum slightly interfered with Arid3a-DNA binding in BCL_1_ but not in M12.4 (Figure 2 and Figure 3). This suggested that the complexes in the two B cell lines are not identical.

Next, we examined the components of each complex by performing super-shift assays with various antibodies. The anti-Arid3a antisera employed has been shown previously [15] to possess no cross-reactivity against CDP/Cux, Arid3b, or any other protein tested. As expected, anti-Arid3a treatment completely ablated the Arid3a complex in BCL_1_, shifting it to the well of the gel (Figure 3). In addition, and unanticipated from previous reports, anti-Arid3a consistently super-shifted the NF-μNR complex formed on either probe to a slightly slower mobility (Figure 2 and Figure 3). This subtle NF-μNR super-shift with anti-Arid3a was also observed in M12.4 cells (Figure 2). The super-shift/ablation of the NF-μNR complex with anti-Cux confirmed that the super-shifted complex with anti-Arid3a is the NF-μNR complex.

**Figure 4:**
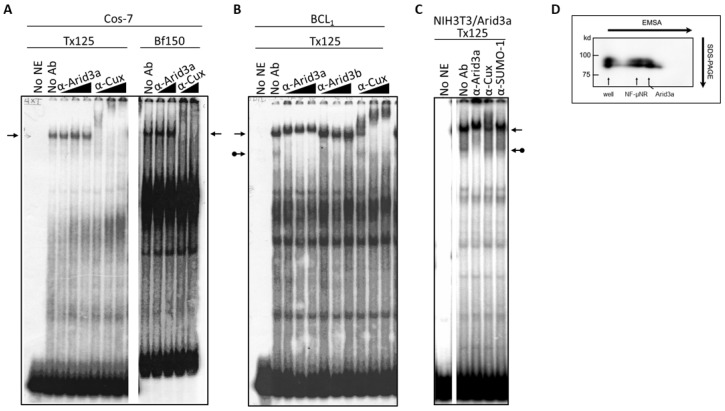
The NF-μNR complex contains Arid3a in B cells but not in non-B cells that were tested. (**A**) NF-μNR in Cos-7 includes CDP/Cux but not Arid3a. Extract and probe preparation, EMSA and supershifts were performed as in Figure 3. Triangles indicate increasing concentration of indicated antibodies. Arrows denote mobility of the NF-μNR complex; (**B**) Anti-Arid3a antiserum super-shifted the NF-μNR complex (plain arrows) as well as the Arid3a complex (arrows with balls) in BCL_1_; (**C**) The NF-μNR complex in NIH3T3 cells (NIH3T3-Arid3a) stably expressing Arid3a via retroviral infection; (**D**) Confirmation of Arid3a within both NF-μNR and Arid3a EMSA complexes by 2-D gel electrophoresis in B cells. First dimension, standard EMSA of nuclear extract prepared from M12.4. Second dimension, SDS-PAGE, was followed by Western blotting with anti-Arid3a. Positions of the two EMSA complexes are indicated by arrows. Molecular markers are shown on the right of the gel.

To further test whether the NF-μNR complex in B cells contains Arid3a, we performed super-shift assays using nuclear extract prepared from Cos-7 cells, which by immunostaining and Western blotting (data not shown) contain no detectable Arid3a protein (Figure 4A). The Cos-7 and BCL_1_ patterns can be compared in Figure 4B. Even though increasing amounts of anti-Arid3a clearly super-shifted the NF-μNR complex in BCL_1_ B cells, it produced no effect on the NF-μNR complex in Cos-7 cells. However, anti-Cux super-shifted the NF-μNR complex in both BCL_1_ and Cos-7.

The presence of Arid3a in the NF-μNR complex in B cells was confirmed by two-dimensional electrophoresis, employing EMSA in the first dimension and SDS-PAGE in the second (Figure 4D). Western blotting with anti-Arid3a detected two spots on the membrane, one corresponding to its mobility within the NF-μNR complex and the other to its mobility within the Arid3a complex (Figure 4D). Therefore, we concluded that Arid3a is a component of NF-μNR in B cells but not in non-B cells.

Next, we addressed whether Arid3a is the only B cell-specific component required for this effect. We utilized a nuclear extract prepared from the Arid3a retrovirally infected stable cell line (NIH3T3-Arid3a; described in Section 2). As shown in Figure 4C, anti-Arid3a ablated/super-shifted both the Arid3a and NF-μNR complexes. This indicated that Arid3a expression *per se* is sufficient for the integration of Arid3a into the NF-μNR complex.

We utilized several antibodies to check the Arid3a and NF-μNR complexes for additional components. Both BCL_1_ and M12.4 also express the B cell-specific Arid protein, Arid3b [20]. Thus, we examined its contribution to the two EMSA complexes using an anti-Arid3b antiserum whose lack of cross-reactivity against either Arid3a or CDP/Cux was previously confirmed (data not shown). As shown in Figure 3 and Figure 4, anti-Arid3b antiserum had no effect on the NF-μNR complex but slightly perturbed the Arid3a complex in BCL_1_. However, anti-Arid3b had no discernible effect on the Arid3a complex in M12.4 cells (Figure 3 and Figure 4).

A previous study [27] indicated that Bruton tyrosine kinase (Btk) interacts with Arid3a and is a component of the Arid3a-DNA complex. However, we were unable to detect super-shifts of either the Arid3a or the NF-μNR complex with a commercially obtained anti-Btk antibody (Figure 2).

A fraction of Arid3a localizes in Hela cells to PML-nuclear bodies [28], and such localization is often accompanied by SUMO-1 post-translational modification. Therefore, we tested whether an anti-SUMO-1 monoclonal antibody (provided by Dr. G. Maul) could super-shift the Arid3a complex. Unexpectedly, anti-SUMO-1 super-shifted the NF-μNR complex rather than the Arid3a complex (Figure 3).

CDP/Cux and Arid3b were reported to interact with Rb [20,29]. Thus, we examined the existence of Rb in the NF-μNR or Arid3a complexes. Anti-Rb antibody did not shift either of the complexes (Figure 3). Negative super-shift controls, including pre-immune (PI) serum and an irrelevant (anti-BCL11) antiserum, had no effect on either complex. These super-shift assays demonstrate an unexpected, previously unappreciated complexity in the NF-μNR “repressive-” and Arid3a “activation-related” complexes formed on the V_H_1 promoter-associated MARs.

### 3.2. DNA Binding Activity of Arid3a Is Sensitive to Cell Cycle and Nuclear Localization

**Figure 5:**
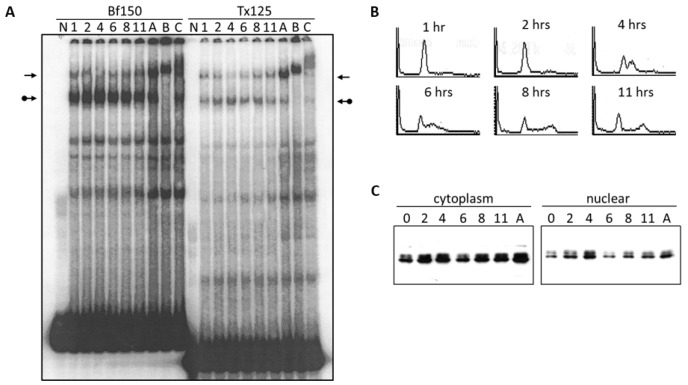
Arid3a binding to promoter-associated MARs increases during S phase. BCL_1_ cell cycle was arrested with 5 μg/mL aphidicolin for 16 h. Cells were washed to release the cell cycle block, cultured for 11 h with aliquots harvested every 2 h (**A**) Nuclei were extracted and EMSA was performed. 0–11 h after cell cycle release, A: asynchronous cells B: super-shift with anti-Arid3a antiserum, C: super-shift with anti-Cux antiserum, N: free probe. Probes are indicated at the top. Plain arrows, NF-μNR, and arrows with balls, Arid3a complexes; (**B**) BCL_1_ cells distribution at each time point based on the DNA content. Cells were fixed with ethanol and stained with propidium iodide. Cell cycle status was examined through FACS analysis; (**C**) Arid3a expression in BCL_1_ cells at each time point after the release of cell cycle block. Extracts were separated on SDS-PAGE and Western blotting was performed. (Loading controls not shown).

We investigated the DNA binding activity of the Arid3a complex and the NF-μNR complex under various conditions such as cell cycle arrest and starvation. Initially, we examined the DNA binding affinity of the complexes after release of BCL_1_ cells from an aphidicolin-arrested cell cycle (see Section 2 for details). Nuclear extracts were prepared at two-hour intervals following release from G_1_/S arrest and were compared by EMSA to extracts prepared from asynchronous cells using Tx125 and Bf150 as probes. Following 11 h after release, the Arid3a complex increased (relative to control) to a maximum at 4 h, while during the same interval, the NF-μNR complex decreased (Figure 5A). The BCL_1_ cell cycle was monitored during the same time course by propidium iodide staining and flow cytometry (Figure 5B). At 1–2 h following release, most cells were still in G_1_. At four hours, about approximately half of the cells remained in G_1_ phase and about half were in S phase. G_2_/M cells began to appear at 6 h, whereas the majority of the cells separated into G_1_ and G_2_/M at ~11 h. The increase in the abundance of the Arid3a complex and the decrease in the NF-μNR complex coincide with the S phase maxima at four hours following release from the cell cycle block.

We next determined the expression levels and the localization of Arid3a in these cells (Figure 5C). After release from cycle block, the expression of Arid3a appeared to increase in both the cytoplasm and the nucleus. However, we consistently observed that the Arid3a levels decreased at the six-hour time point in both compartments, followed by a gradual, continued increase after 11 h. In addition, the nuclear/cytoplasmic ratio of Arid3a modestly increased at 4 h, post arrest, compared to the ratio in the asynchronous culture. Therefore, DNA binding activity of the Arid3a complex appeared to increase as a result from its increase in nuclear abundance. The data indicate that this increase may be achieved by increased Arid3a transcription, increased nuclear entry, decreased nuclear export or by both mechanisms.

### 3.3. LPS Stimulates Formation of the NF-μNR Complex

**Figure 6:**
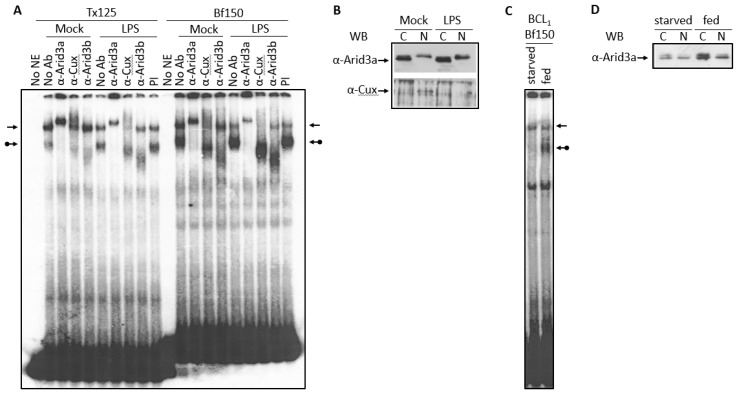
Increased Arid3a expression correlated with the increased Arid3a-DNA interaction. (**A**,**B**) Nuclei of LPS- or mock-treated BCL_1_ cells were extracted. (**A**) The nuclear extract was subjected to EMSA with Bf150 probe and Tx125 probe. The arrows indicate the NF-μNR complex and the arrows with a ball indicate the Arid3a complex; (**B**) 2 μg or 5 μg of extract were separated on SDS-PAGE and Western blotting was performed using anti-Arid3a antiserum or anti-Cux antiserum; (**C**,**D**) BCL_1_ cells were grown with or without fresh media supplement for 3 days. Nuclei were extracted (**C**) The nuclear extract was subjected to EMSA with Bf150 probe; (**D**) 2 μg of extract was separated on SDS-PAGE and Western blotting was performed with anti-Arid3a antiserum.

Normal B cells and the BCL_1_ cell line respond to the B cell mitogen LPS by differentiating to a higher antibody secretion state [9]. Thus, we examined the effect of LPS on the abundance of the Arid3a and the NF-μNR EMSA complexes. In BCL_1_ cells treated with LPS for 2 days, we observed an increase in the Arid3a complex on both Bf150 and Tx125 MARs, whereas the NF-μNR complex decreased (Figure 6A). However, no changes were observed in anti-Arid3a, anti-Cux or anti-Arid3b super-shift patterns in either mock-treated or LPS-treated cells (Figure 6A). This prompted an examination of the expression and the localization of Arid3a and Cux within these cells. As shown in Figure 6B, LPS-treated cells expressed more Arid3a, but the nuclear/cytoplasmic ratio of Arid3a was unaffected. On the other hand, Cux accumulated only within the nucleus, and LPS treatment reduced its expression level there. We conclude that these mitogen-induced reciprocal changes in Arid3a and NF-μNR DNA can be attributed to the concomitant changes in the nuclear abundance of Arid3a and Cux/CDP.

### 3.4. Increased Nuclear Levels of Arid3a Is Sufficient for High Affinity Arid3a-MAR Complex Formation

Starvation of cultured mammalian cells results in a mid-G_1_ arrest (quiescence) that differs mechanistically from G_1_/S or G_0_ arrest (senescence) [30]. To determine the potential effect of starvation on Arid3a DNA binding, BCL_1_ cells were allowed to reach confluence by growing them for 3 days without media supplementation. Nuclear extracts were prepared and subjected to EMSA. As shown in Figure 6C, starvation abolished the Arid3a-DNA complex and slightly increased the NF-μNR complex. In starved cells, Arid3a expression was reduced as compared with that in regularly supplemented cells. However, there was no significant change in the nuclear/cytoplasmic ratio of Arid3a (Figure 6D).

**Figure 7:**
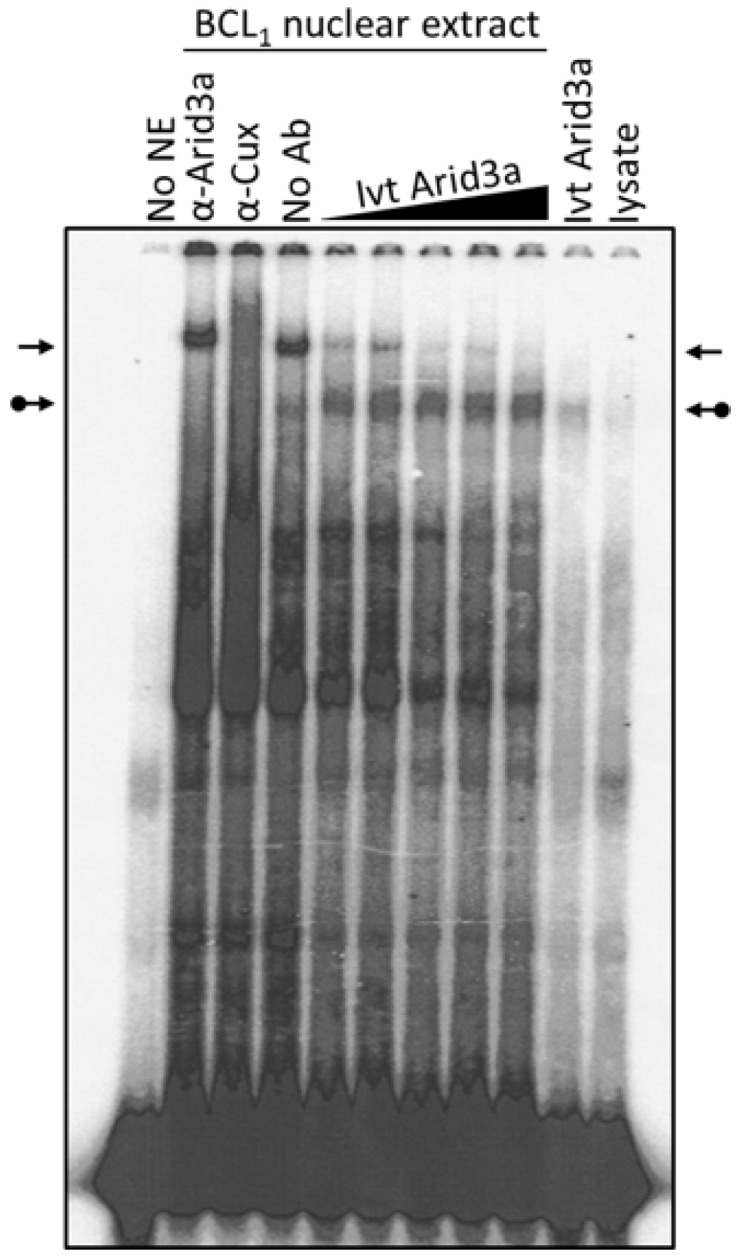
Arid3a over-expression abrogates NF-μNR-DNA binding. BCL_1_ nuclei were extracted and the extract was mixed with in vitro translated Arid3a before DNA binding. The mixture was subject to EMSA using Tx125 probe. The unmodified arrows indicate the NF-μNR complex and the dotted arrows indicate the Arid3a complex.

Cell cycle arrest, LPS stimulation and starvation experiments together indicated that increased nuclear Arid3a expression correlates with increased abundance of the Arid3a-promoter MAR EMSA complex and reduced abundance of the NF-μNR complex assembled on the promoter MARs. Therefore, we asked whether merely increasing the levels of Arid3a is sufficient by performing a simple mixing experiment (Figure 7). Fixed amounts of BCL_1_ nuclear extract were mixed with increasing amounts of in vitro translated Arid3a, and EMSAs were performed using Tx125 as probe. Increasing in vitro translated Arid3a abrogated the NF-μNR complex while increasing the Arid3a complex (Figure 7). Therefore, we suggest that an increased amount of Arid3a in the nucleus may be all that is required for high affinity Arid3a-MAR complex formation.

### 3.5. Transactivation Activity of Arid3a in Non-B Cells Is Modulated by Promoter-Associated MARs

**Figure 8:**
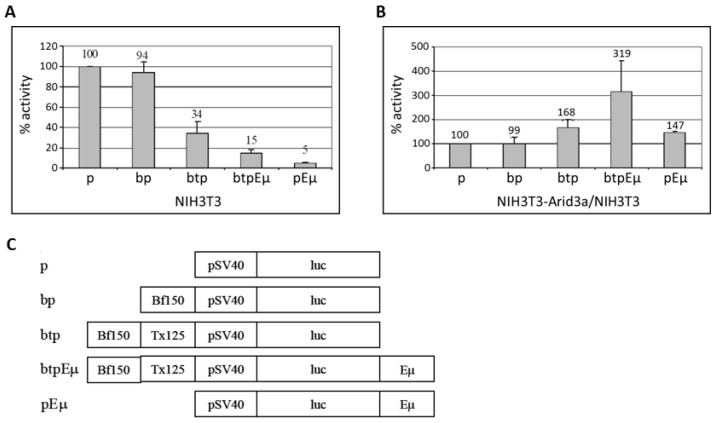
Arid3a transactivates the core SV40 promoter in non-B cells through promoter-associated (5’) MARs (Bf150 and Tx125). NIH3T3 cells and NIH3T3-Arid3a cells (which express retroviral Arid3a) were transfected with firefly luciferase vectors containing Bf150, 5’ MARs (Bf150 + Tx125), Eμ or 5’ MAR + Eμ and Renilla luciferase as control. At 24 h after transfection, cell lysates were prepared and the luciferase activities were measured and normalized using the dual luciferase system. (**A**) 5’ MARs repress SV40 promoter-driven transcription in NIH3T3 cells. Values are plotted relative to 100% for the control (construct p); (**B**) Arid3a transactivates the SV40 core promoter through 5’ MARs and/or Eμ in NIH3T3-Arid3a. Values are obtained from a NIH3T3-Arid3a cell line as above and divided by the values (shown in **A**) obtained from NIH3T3 and plotted relative to 100% for the control. Values were obtained from three independent experiments. (**C**) Graphical summary of reporter gene constructs used.

A previous report demonstrated that a rearranged S107 IgH transgene driven by the V_H_1 core promoter (−125 bp upstream) devoid of MAR regions is not expressed in transgenic non-lymphoid cells [12]. The authors concluded from these data that sequences −125 bp upstream of the V_H_1 transcription start site are sufficient for the repression of an IgH gene in non-B cells. However, that result in no way formally excluded the possibility that the promoter-associated MARs upstream to −125 repress non-B IgH transcription in the absence of an enhancer; i.e., the situation achieved in “germline” targeting of rearrangement to a particular V_H_ segment [9]. To test the effect of the promoter-associated MARs (5’ MARs), we generated firefly luciferase (pGL3-promoter) vectors containing Bf150 (pGL3bfp) and Bf150 plus Tx125 (pGL3btp) upstream of the SV40 minimal core promoter (Figure 8). To test the effect of the enhancer, we constructed Eμ containing reporter plasmids (pGL3pEμ and pGL3btpEμ) in which Eμ was inserted downstream of the poly-A signal of the reporter gene. These were then transiently transfected into NIH3T3 with each of the pGL3 firefly luciferase reporter plasmids along with pRL-Renilla as loading controls. After 48 h, the luciferase activity was measured and the transactivation activity was analyzed. As shown in Figure 8A, Bf150 plus Tx125 (5’ MARs) as well as Eμ and 5’ MARs or Eμ alone repressed reporter gene transcription in NIH3T3 cells to levels varying from ~3–20 fold, while Bf150 alone had no effect. These data indicated that Bf150 plus Tx125 may mediate the repression of IgH gene transcription in non-B cells. However, a significant de-repression (~7 fold) of the Eμ-mediated effect was detected when Eμ and 5’ MARs were positioned upon the same construct.

To examine the effect of Arid3a on these regulatory elements, we performed the same transfections in NIH3T3-Arid3a cells (NIH3T3 cells stably expressing retroviral Arid3a). While a similar pattern of repression was observed, the magnitudes of several of the reporters were significantly reduced, indicative of specific de-repression by Arid3a (data not shown). To best illustrate this, we compared the luciferase activities obtained in NIH3T3 to those from NIH3T3-Arid3a cells (Figure 8B). Arid3a expression increased the basal level transcription through the SV40 minimal promoter. As expected from the previous results, Arid3a had no effect on the luciferase activity mediated through Bf150 when basal level increase was compensated. However, Arid3a expression moderately increased the reporter gene transcription mediated through 5’ MARs, Eμ-only or both elements combined. As we previously observed in B cells [10], the highest levels of transactivation activity were achieved when the 5′ MARs and Eμ together flank the core promoter on the same vector (Figure 8B).

Collectively, these experiments indicate that Arid3a can transactivate IgH gene transcription through linked 5′ MARs and Eμ in non-B cells by de-repressing the locus.

## 4. Discussion

Tissue-specific transcription of a progressively rearranged IgH gene has been shown to require a core V_H_ promoter and the intronic enhancer Eμ [7]. Eμ has been shown to be sufficient for the repression of IgH transcription in non-B cells. In this study, we showed that the V_H_1 promoter-associated MARs, Bf150 and Tx125, also can repress reporter gene transcription in non-B cells in a concerted fashion and, further, they can relieve the repression mediated by Eμ in non-B cells. Arid3a transactivated reporter gene transcription through Eμ or the 5’ MARs. Arid3a transactivation was maximal when Eμ and the 5’ MARs flanking the promoter were positioned on the same construct. This is a context analogous to that following VDJ rearrangement, in which the promoter and Eμ are juxtaposed to function synergistically for IgH gene transcription in B cells.

Bf150 and Tx125 are occupied by the NF-μNR and Arid3a complexes in B cells, but only by the NF-μNR complex in non-B cells. Here, we showed that Arid3a is a component of the NF-μNR complex as well as the Arid3a complex in B cells. Arid3a, when assembled into the NF-μNR complex, may confer different function to this complex in B cells and non-B cells. One hypothesis is that Arid3a may relieve the repression mediated by NF-μNR—an idea supported by the transactivation data of Figure 8. Another possibility is that Arid3a may weaken the NF-μNR-DNA interaction and facilitate rapid dissociation of the complex in B cells, particularly in response to stimulation with agents such as LPS. Because Arid3a expression, in itself, was sufficient for its incorporation into the NF-μNR-DNA complex in non-B cells, Arid3a participation may be prerequisite for the de-repression of IgH prior to the time in which it might actively accelerate transcriptional initiation in a positive manner.

Our EMSA super-shift data indicated that the NF-μNR complex contains SUMO-1 modified protein(s) both in B and non-B cells. In many cases, SUMO-1 modification is required for sub-nuclear localization such as within the PML-nuclear body [31]. This raises the possibility that the promoter-associated MARs, when bound by NF-μNR, may become sequestered into a restricted, heterochromatin region, such as the PML body, in non-B cells. This would lead to a repressed state of IgH. Indeed, several studies have shown that genes can change their nuclear neighborhoods by re-positioning from repressive sites to transcriptionally active compartments, and vice versa [32,33,34,35]. The nuclear positionings of Ig loci also are tightly regulated, as they are positioned centrally in committed B-cell progenitors, while in non-B cells they are located at the nuclear periphery [35,36,37]. It is conceivable that Arid3a, via its ability to condense chromatin and partition nucleosomes, might contribute to such relocalization. Re-localization of the IgH locus from a sequestered nuclear area would facilitate the access of other transcription factors to the locus.

The Arid3a EMSA complex also includes Arid3b. Previously, we showed that Arid3a binds Arid3b, a paralog of Arid3a, through a REKLES domain in vitro [22]. Therefore, we reasoned that Arid3b may exist in a form of homomers or Arid3a-Arid3b heteromers in the Arid3a/DNA complex. From the data of Figure 2 and Figure 3, it would appear that most of the Arid3a complex in BCL_1_ is composed of Arid3a-Arid3b heteromers, because both anti-Arid3a and anti-Arid3b quantitatively super-shift the complex. However, while Arid3b and CDP/Cux may be recruited to the Arid3a complex in BCL_1_, this does not appear to be the case in M12.4 B cells. Nor does either B cell line appear to contain Arid3b in the NF-μNR complex. These results suggest that the differential composition of the Arid3a complex may dictate differential functions or activities for Arid3a during B cell development and differentiation. However, we were unable to confirm previous results that predict that both Rb and Btk participate in the Arid3a and NF-μNR complexes, respectively [20,27]. It is unclear at this time whether this is a technical problem, such as gel or antibody conditions, a problem of cell heterogeneity or whether neither actually participates in the complexes. Additional studies are needed to resolve this issue.

Wang et al. demonstrated that over-expression of CDP, a component of NF-µNR, abrogated Arid3a-MAR DNA interaction [15]. Conversely, we found that Arid3a over-expression can abrogate NF-μNR MAR DNA binding. This suggests a straightforward model in which the relative amount of Arid3a and NF-μNR available within the correct nuclear location determines the relative DNA binding activity of Arid3a and NF-μNR complexes. Transcription of the genes for the human histone proteins are activated at G_1_/S phase transition and maintained during S phase by HiNF-D, whose functional core component is Cux. Cux interacts with cell cycle control elements not only within HiNF-D but singularly in other systems [29]. We induced cell cycle arrest at G_1_/S using the DNA-polymerase α inhibitor, aphidicolin. Following release, BCL_1_ cells showed decreased DNA binding of NF-μNR and increased DNA binding of Arid3a. HiNF-D and NF-µNR consist of different components even though CDP/Cux is found in both. These differences may underlie the disparity of the DNA binding properties of the two complexes. The enhanced Arid3a DNA binding we observed at four hours following release appears to result directly from increased Arid3a expression. In addition, starvation decreased Arid3a expression and Arid3a-DNA binding but increased NF-μNR-DNA binding. Increased Arid3a expression following LPS stimulation in B cells also correlated with increased DNA binding of Arid3a and reduced DNA binding of NF-μNR. Therefore, we conclude that the Arid3a-NF-μNR ratio in the nucleus plays a crucial role in regulating IgH gene transcription in B cells and its repression in non-B cells.

IgH gene transcription is regulated in a highly complicated manner. This process is controlled by a variety of transcription factors, and many of them, including Arid3a, are subject to regulation by their tissue specificity, development/differentiation stage specificity and extracellular stimulation. For example, LPS or IL-5 and antigen induced the differentiation of BCL_1_ cells to plasmablasts/plasma cells specialized for the secretion of high levels of antibodies [38,39] and increased Arid3a-MAR binding (Figure 6A) [40]. The V_H_1 promoter-associated 5′ MARs, Bf150 and Tx125, were elucidated here as elements involved in the regulation of IgH gene transcription.

**Figure 9:**
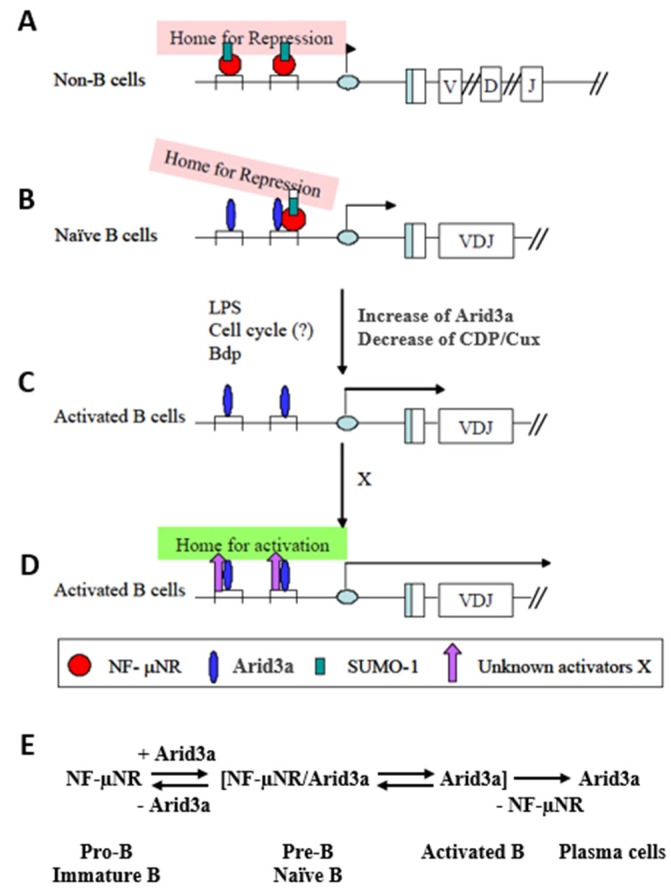
Hypothetical function of Arid3a in regulating IgH transcription through 5′ MARs. (**A**) IgH V_H_-associated promoter region may be sequestered within a sub-nuclear region(s) in which most transcription activities are repressed in non-B cells; (**B**) The interaction of Arid3a with CDP/Cux may change the DNA binding affinity of the NF-µNR complex and/or increase the accessibility of other components which may comprise the Arid3a complex. These changes in NF-µNR may alter the complex such that it can dissociate from MARs; (**C**) Activation of the B cells increases the affinity of Arid3a for both MARs, allowing the IgH promoter to be liberated from the repressive region by displacement of NF-µNR; (**D**) As cells become more activated and move toward terminal differentiation, the Arid3a complex may change its components so as to re-localize the IgH promoter to a euchromatic sub-nuclear region; (**E**) Hypothesized equilibrium of Arid3a and NF-µNR during B cell development.

We have summarized our data in the model of Figure 9. When occupied by NF-μNR, the MARs function to repress IgH in non-B cells. From the presence of SUMO-1 in this complex, we hypothesized that the IgH V_H_-associated promoter region may be sequestered within a subnuclear region(s) in which most transcription activities are repressed, such as within nuclear dots (Figure 9A). In B cells, the 5’ MARs can be bound by the NF-μNR and/or the Arid3a complex. The equilibrium between these complexes depends on the relative expression levels of Arid3a and CDP/Cux. These levels are controlled in a dominant way as Arid3a expression levels increase. But as Arid3a expression levels rise (e.g., in the transition from a pre-B cell to a mature B cell or following mitogenic stimulation of mature B cells), Arid3a partitions into both the NF-μNR complex and the Arid3a complex. The interaction of Arid3a with NF-μNR may change the DNA binding affinity of the NF-μNR complex and/or increase the accessibility of other components which may comprise the complex. These changes in NF-μNR may alter the complex such that it can dissociate from the MARs (Figure 9B). In addition, the two MARs appear to have different affinities for the NF-μNR complex and the Arid3a complex. In unstimulated cells, Tx125 has high affinity for NF-μNR, while Bf150 has high affinity for the Arid3a complex. This could allow the IgH promoter to associate with putative repressive sub-nuclear regions more loosely in B cells than in non-B cells. Finally, we suggest that activation of B cells increases the affinity of Arid3a for both MARs, allowing the IgH promoter to be liberated from the repressive region by displacement of NF-μNR (Figure 9C). As cells become more activated and move toward terminal differentiation, the Arid3a complex may change its components so as to re-localize the IgH promoter to a euchromatic sub-nuclear region.

## Supplementary Materials

The following are available online. Figure S1: Intronic (Eμ) and 3′ enhancers within the IgH locus. (A) Eμ is composed of a core enhancer and two flanking MAR regions. MAR regions contain four A/T-rich P-site protein binding motifs (hatched boxes) and the core enhancer contains several motifs (open boxes). Transcription factors are shown under the motifs to which they bind; (B) IgH 3′ enhancer is comprised of four DNase I hypersensitive sites (open boxes and arrows). HS3a and HS4 are inverted repeats that form palindrome structures; (C) Position of MARs within the rearranged IgH locus. Closed ovals indicate MARs; open ovals, enhancers; open boxes, exons.

## References

[1] Wasylyk C., Wasylyk B. (1986). The immunoglobulin heavy-chain B-lymphocyte enhancer efficiently stimulates transcription in non-lymphoid cells. EMBO J..

[2] Matthias P., Baltimore D. (1993). The immunoglobulin heavy chain locus contains another B-cell-specific 3’ enhancer close to the alpha constant region. Mol. Cell. Biol..

[3] Chauveau C., Jansson E.A., Muller S., Cogne M., Pettersson S. (1997). Cutting edge: Ig heavy chain 3’ HS1–4 directs correct spatial position-independent expression of a linked transgene to B lineage cells. J. Immunol..

[4] Le Noir S., Boyer F., Lecardeur S., Brousse M., Oruc Z., Cook-Moreau J., Denizot Y., Cogné M. (2017). Functional anatomy of the immunoglobulin heavy chain 3’ super-enhancer needs not only core enhancer elements but also their unique DNA context. Nucleic Acids Res..

[5] Cockerill P.N., Yuen M.H., Garrard W.T. (1987). The enhancer of the immunoglobulin heavy chain locus is flanked by presumptive chromosomal loop anchorage elements. J. Biol. Chem..

[6] Banerji J., Olson L., Schaffner W. (1983). A lymphocyte-specific cellular enhancer is located downstream of the joinings region in immunoglobulin heavy chain genes. Cell.

[7] Staudt L.M. (1991). Immunoglobulin gene transcription. Annu. Rev. Immunol..

[8] Perez-Mutul J., Macchi M., Wasylyk B. (1998). Mutational analysis of the contribution of sequence motifs within the IgH enhancer to tissue specific transcriptional activation. Nucleic Acids Res..

[9] Webb C.F., Zong R.T., Lin D., Wang Z., Kaplan M., Paulin Y., Smith E., Probst L., Bryant J., Goldstein A. (1999). Differential regulation of immunoglobulin gene transcription via nuclear matrix-associated regions. Cold Spring Harb. Symp. Quant. Biol..

[10] Kaplan M.H., Zong R.T., Herrscher R.F., Scheuermann R.H., Tucker P.W. (2001). Transcriptional activation by a matrix associating region-binding protein. Contextual requirements for the function of bright. J. Biol. Chem..

[11] Herrscher R.F., Kaplan M.H., Lelsz D.L., Das C., Scheuermann R., Tucker P.W. (1995). The immunoglobulin heavy-chain matrix-associating regions are bound by Bright: A B cell-specific trans-activator that describes a new DNA-binding protein family. Genes Dev..

[12] Avitahl N., Calame K.A. (1996). 125 bp region of the Ig VH1 promoter is sufficient to confer lymphocyte-specific expression in transgenic mice. Int. Immunol..

[13] Grosschedl R., Weaver D., Baltimore D., Costantini F. (1988). Introduction of a mu immunoglobulin gene into the mouse germ line: Specific expression in lymphoid cells and synthesis of functional antibody. Cell.

[14] Aguilera R.J., Hope T.J., Sakano H. (1985). Characterization of immunoglobulin enhancer deletions in murine plasmacytomas. EMBO J..

[15] Wang Z., Goldstein A., Zong R.T., Lin D., Neufeld E.J., Scheuermann R.H., Tucker P.W. (1999). Cux/CDP homeoprotein is a component of NF-μNR and represses the immunoglobulin heavy chain intronic enhancer by antagonizing the Bright transcription activator. Mol. Cell. Biol..

[16] Neufeld E.J., Skalnik D.G., Lievens P.M., Orkin S.H. (1992). Human CCAAT displacement protein is homologous to the Drosophila homeoprotein, cut. Nat. Genet..

[17] Li S., Moy L., Pittman N., Shue G., Aufiero B., Neufeld E.J., LeLeiko N.S., Walsh M.J. (1999). Transcriptiona l repression of the cystic fibrosis transmembrane conductance regulator gene, mediated by CCAAT displacement protein/cut homolog, is associated with histone deacetylation. J. Biol. Chem..

[18] Coqueret O., Berube G., Nepveu A. (1998). The mammalian Cut homeodomain protein functions as a cell-cycle-dependent transcriptional repressor which downmodulates p21WAF1/CIP1/SDI1 in S phase. EMBO J..

[19] Van Gurp M.F., Pratap J., Luong M., Javed A., Hoffmann H., Giordano A., Stein J.L., Neufeld E.J., Lian J.B., Stein G.S. (1999). The CCAAT displacement protein/cut homeodomain protein represses osteocalcin gene transcription and forms complexes with the retinoblastoma protein-related protein p107 and cyclin A. Cancer Res..

[20] Numata S., Claudio P.P., Dean C., Giordano A., Croce C.M. (1999). Bdp, a new member of a family of DNA-binding proteins, associates with the retinoblastoma gene product. Cancer Res..

[21] Kim D., Tucker P.W. (2006). A regulated nucleocytoplasmic shuttle contributes to Bright’s function as a transcriptional activator of immunoglobulin genes. Mol. Cell. Biol..

[22] Kim D., Probst L., Das C., Tucker P.W. (2007). REKLES is an ARID3-restricted multifunctional domain. J. Biol. Chem..

[23] Laskov R., Kim J.K., Woods V.L., McKeever P.E., Asofsky R. (1981). Membrane immunoglobulins of spontaneous B-lymphomas of aged BALB/c mice. Eur. J. Immunol..

[24] Gronowicz E.S., Doss C.A., Howard F.D., Morrison D.C., Strober S. (1980). An in vitro line of the B cell tumor BCL1 can be activated by LPS to secrete IgM1. J. Immunol..

[25] Johnson D.R., Levanat S., Bale A.E. (1995). Isolation of intact nuclei for nuclear extract preparation from a fragile B-lymphocyte cell line. Bio-Techniques.

[26] Webb C.F., Das C., Eneff K.L., Tucker P.W. (1991). Identification of a matrix associated region 5’ of an immunoglobulin heavy chain variable region gene. Mol. Cell. Biol..

[27] Webb C.F., Yamashita Y., Ayers N., Evetts S., Paulin Y., Conley M.E., Smith E.A. (2000). The transcription factor Bright associates with Bruton’s tyrosine kinase, the defective protein in immunodeficiency disease. J. Immunol..

[28] Zong R.T., Das C., Tucker P.W. (2000). Regulation of matrix attachment region dependent, lymphocyte-restricted transcription through differential localization within promyelocytic leukemia nuclear bodies. EMBO J..

[29] Van Wijnen A.J., van Gurp M.F., de Ridder M.C., Tufarelli C., Last T.J., Birnbaum M., Vaughan P.S., Giordano A., Krek W., Neufeld E.J. (1996). CDP/cut is the DNA-binding subunit of histone gene transcription factor HiNF-D: A mechanism for gene regulation at the G1/S phase cell cycle transition point independent of transcription factor E2F. Proc. Natl. Acad. Sci. USA.

[30] Ho A., Dowdy S.F. (2002). Regulation of G1 cell-cycle progression by oncogenes and tumor suppressor genes. Curr. Opin. Genet. Dev..

[31] Ishov A.M., Sotnikov A.G., Negorev D., Vladimirova O.V., Neff N., Kamitani T., Yeh E.T., Strauss J.F., Maul G.G. (1999). PML is critical for ND10 formation and recruits the PML- interacting protein daxx to this nuclear structure when modified by SUMO-1. J. Cell Biol..

[32] Schneider R., Grosschedl R. (2007). Dynamics and interplay of nuclear architecture, genome organization, and gene expression. Gene Dev..

[33] Osborne C.S., Chakalova L., Brown K.E., Carter D., Horton A., Debrand E., Goyenechea B., Mitchell J.A., Lopes S., Reik W. (2004). Active genes dynamically colocalize to shared sites of ongoing transcription. Nat. Genet..

[34] Peric-Hupkes D., Meuleman W., Pagie L., Bruggeman S.W., Solovei I., Brugman W., Graf S., Flicek P., Kerkhoven R.M., van Lohuizen M. (2010). Molecular maps of the reorganization of genome-nuclear lamina interactions during differentiation. Mol. Cell.

[35] Reddy K.L., Zullo J.M., Bertolino E., Singh H. (2008). Transcriptional repression mediated by repositioning of genes to the nuclear lamina. Nature.

[36] Kosak S.T., Skok J.A., Medina K.L., Riblet R., Le Beau M.M., Fisher A.G., Singh H. (2002). Subnuclear compartmentalization of immunoglobulin loci during lymphocyte development. Science.

[37] Skok J.A., Brown K.E., Azuara V., Caparros M.L., Baxter J., Takacs K., Dillon N., Gray D., Perry R.P., Merkenschlager M. (2001). Nonequivalent nuclear location of immunoglobulin alleles in B lymphocytes. Nat. Immunol..

[38] Lafrenz D., Koretz S., Stratte P.T., Ward R.B., Strober S. (1982). LPS-induced differentiation of a murine B cell leukemia (BCL1): Changes in surface and secreted IgM. J. Immunol..

[39] Brooks K., Yuan D., Uhr J.W., Krammer P.H., Vitetta E.S. (1983). Lymphokine-induced IgM secretion by clones of neoplastic B cells. Nature.

[40] Webb C.F., Das C., Coffman R.L., Tucker P.W. (1989). Induction of immunoglobulin µ mRNA in a B cell transfectant stimulated with interleukin-5 and T-dependent antigen. J. Immunol..

