# Supplementary material for: Competitive Promoter-Associated Matrix Attachment Region Binding of the Arid3a and Cux1 Transcription Factors
Source: Diseases. 2017 Dec 10;5(4):34. doi: 10.3390/diseases5040034 (PMC5750545; doi:10.3390/diseases5040034)
Supplement: Supplementary file 1 [file diseases-05-00034-s001.pdf]

Supplementary Materials: Competitive Promoter-Associated Matrix Attachment Region Binding of the Arid3a and Cux1 Transcription Factors

Dongkoom Kim, Christian Schmidt, Mark A. Brown and Haley Tucker

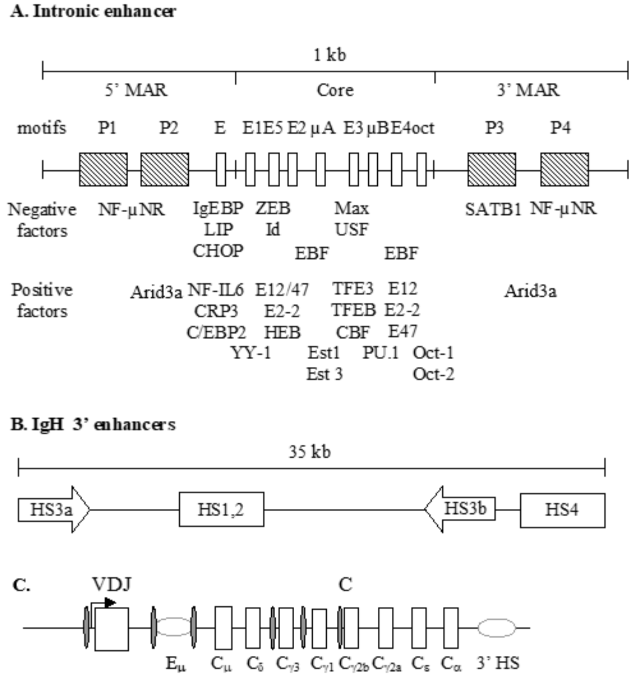

Figure S1. Intronic (Eμ) and 3′ enhancers within the IgH locus. (**A**) Eμ is composed of a core enhancer and two flanking MAR regions. MAR regions contain four A/T-rich P-site protein binding motifs (hatched boxes) and the core enhancer contains several motifs (open boxes). Transcription factors are shown under the motifs to which they bind. (**B**) IgH 3′ enhancer is comprised of four DNase I hypersensitive sites (open boxes and arrows). HS3a and HS4 are inverted repeats that form palindrome structures. (**C**) Position of MARs within the rearranged IgH locus. Closed ovals indicate MARs; open ovals, enhancers; open boxes, exons.
